# Totally extraperitoneal repair of inguinal hernia: A case for bilateral repair

**DOI:** 10.4103/0972-9941.27733

**Published:** 2006-09

**Authors:** Pradeep K Chowbey, Murtaza Pithawala, Rajesh Khullar, Anil Sharma, Vandana Soni, Manish Baijal

**Affiliations:** Minimal Access and Bariatric Surgery Centre, Sir Ganga Ram Hospital, New Delhi - 110 060, India

**Keywords:** Bilateral, collagen, occult hernia, total extraperitoneal repair

## Abstract

Inguinal hernia surgery has been one of the most extensively debated and continues to evolve in search for the ideal technique. Even though the method to diagnose hernia has largely remained clinical, recently other modalities have detected hernias that are not picked up on clinical examination or are incorrectly labeled. Laparoscopy, for the first time has given surgeons the unique opportunity to look at both sides of the groin and to detect and study the contra lateral groin in a patient of clinically unilateral hernia. This has given rise to some interesting findings. In the pediatric age group the value of bilateral detection and repair has been extensively debated. However, the same is not true for the adults despite the facts that there are better methods for detection, better understanding of pathogenesis of hernia and better repair techniques that can take care of bilateral repair without adding to morbidity.

That hernia is not a simple derivative of patent processus vaginalis or strain related cause is beginning to be better understood now. It may primarily be a disorder of collagen metabolism with genetic basis. Laparoscopy has also made us wiser in detection of type of hernia and examination of both groin areas. In several studies there have been a high percentage of undetected hernias or additional defects. This was never realized earlier as in open surgery there is no question of exploring the asymptomatic groin. Laparoscopy in bilateral repair is safe and does not add significantly to the operating time, cost or morbidity.

At our Department of Minimal Access Surgery, Sir Ganga Ram Hospital, New Delhi, India we have been performing Endoscopic Totally Extraperitoneal (TEP) repair for all simple and complicated inguinal hernia since 1994. We now routinely perform a bilateral repair based on our understanding that the pathogenesis of hernia is a complex process and any genetic basis of collagen disorder has to affect the patient bilaterally. The clinical examination may have unacceptably low sensitivity. Early identification and repair obviates the need for reoperation, reduces overall costs and eliminates further anaesthetic and operative risks for the patient

Endoscopic hernia repair has been an important addition in the armamentarium of surgeons worldwide and has resulted in offering a less morbid, less painful and more patient-friendly repair to the patient. Several studies have reported simultaneous repair of occult bilateral hernias with added advantage to patients. In view of recent reports of an undergoing collagen metabolism disorder playing a role in pathogenesis, it is likely that inguinal hernias are a bilateral occurrence over course of time. Herein we present our case for simultaneous repair of bilateral inguinal hernias using the endoscopic totally extraperitoneal approach.

## PATHOGENESIS

Conventional teaching believes the etiopathogenesis of inguinal hernias to be an abnormal increase in intra-abdominal pressure. This may occur due to lifting of heavy weights, chronic cough, chronic constipation, urinary outflow obstruction, etc. A patent processus vaginalis was termed to be another important factor, especially in the etiology of congenital hernias.

Studies done before 1993 had shown that alterations in the connective tissue accumulation might have played a role in the development of hernia. Imperfect collagen synthesis disorders such as osteogenesis imperfecta are known to develop an increased incidence of herniations.

The tensile strength of a tissue is largely dependent on the varying proportions of type I collagen, with its high tensile strength and immature collagen type 3. This relationship is regulated by several matrix metalloproteinases, especially 1 and 13. Fibronectin plays a key role in the adherence of cells within the extracellular matrix.

An increase in type 3 collagen may result in reduced collagen fibril assembly in the abdominal wall, leading to development of hernia.[1] In another study, it was stated that the ratio of type 1 / type 3 collagen was markedly reduced in the hernial sac of patients with inguinal hernia.[2] In recurrent hernias, these patients were found to have an increase in both procollagen type 3 mRNA and subsequent type 3 collagen synthesis as well as increase in matrix metalloproteinases 1 and 13.[3] This strongly suggests that recurrent inguinal hernias could be a disease of the collagen matrix. Thus the knowledge of transcriptional regulation of collagen in patients with inguinal hernia may help to understand its pathogenesis and help in devising new therapeutic strategies for this disease.

## DETECTION AND DIAGNOSIS

The diagnosis of hernia is made on clinical examination and is likely to be influenced by the experience of the surgeon. Hence it has a high rate of false negativity and positivity. Many a hernias may be missed, especially early hernias. Newer investigative modalities like USG, MRI and laparoscopy have helped in early detection of occult hernias and simultaneous repair of these, thereby preventing the additional morbidity of a second procedure. Many studies have demonstrated the role of laparoscopy in identifying undiagnosed contralateral hernias, simultaneous repair and the high rates of success and safety in laparoscopic hernia repairs even in community hospitals. Crawford DL *et al* studied 253 patients over a period of 7 years.[4] TEP was performed in 93% of the 560 hernia repairs. A diagnostic laparoscopy revealed that 50 of the 73 patients diagnosed for unilateral hernia had a wrong diagnosis and 37 of these had bilateral hernia. Another study by Woodward AM *et al* found that 64% of patients were found to have a ‘secondary hernia’ not detected on clinical examination.[5] These studies have shown that laparoscopy offers a safe and feasible surgical option for hernia repair with an added advantage of identifying bilateral and occult hernias. This may be responsible for a lower recurrence rate than might have been expected otherwise.[5]

Another study done by Sayad P *et al* showed that 26% of contralateral hernias were diagnosed only at the time of surgery.[6] They also concluded that the time taken for contralateral exploration was 2–5 min and that there was no additional morbidity after a negative exploration.

## OCCULT CONTRALATERAL HERNIAS

The other question that arises is whether to repair the defects seen on contralateral exploration, i.e, occult asymptomatic contralateral hernias. A study done by V. K. Thumbe *et al* showed that over a period of 12 months, 28% of occult hernias developed symptoms necessitating a repair[7] and at surgery, it was seen that many of these had enlarged significantly.

## UNILATERAL VERSUS BILATERAL REPAIR

This question has plagued the minds of eminent endoscopic surgeons worldwide and has generated intense debate. In a study done by Schmedt CG,[8] data were analyzed from 5,524 consecutive patients who underwent laparoscopic hernia repair at a single center, out of which 1,336 patients had bilateral repair done and a unilateral repair in the rest (4,188).[8] Morbidity and reoperation rates showed no statistically significant difference between the two groups. On a median follow-up of 24 months (1-84 months), the recurrence rate was 0.9% for unilateral and 0.6% for bilateral. Median work off was 14 days for unilateral and 17 days for bilateral. This study concluded that bilateral repair was safe, comfortable for patients, cost-effective, without increased morbidity and recurrence risk.

Another study done by Hung Lau *et al* also concluded that postoperative morbidity and recovery were equivalent for those who underwent bilateral repair when compared to unilateral repair.[9]

## CONCLUSIONS

The pathogenesis of inguinal hernia is a complex process not yet fully understood. Genetic regulation of collagen synthesis plays an important role in both primary and recurrent inguinal hernias. Clinical examination has a low sensitivity and high false positive and negative rates. Laparoscopy awards the surgeon the unique advantage of detecting occult contralateral hernias, which should be repaired during the same surgery. Doing so does not in any way increase the operative time, cost or morbidity to the patient. In fact, early identification and repair obviates the need for reoperation with its added costs and eliminates further anesthetic and operative risks and loss of work for the patient.
